# Expert opinion on current and future prophylaxis therapies aimed at improving protection for people with hemophilia A

**DOI:** 10.25122/jml-2022-0103

**Published:** 2022-04

**Authors:** Angelika Batorova, Ana Boban, Melen Brinza, Toshiko Lissitchkov, Laszlo Nemes, Irena Zupan Preložnik, Petr Smejkal, Nadezhda Zozulya, Jerzy Windyga

**Affiliations:** 1. Department of Hematology and Transfusion Medicine, National Hemophilia Center, Faculty of Medicine of Comenius University and University Hospital, Bratislava, Slovakia; 2. Haemophilia Centre, University Hospital Centre Zagreb, School of Medicine, University of Zagreb, Zagreb, Croatia; 3. Centre of Hematology and Bone Marrow Transplant, European Haemophilia Comprehensive Care Centre, Fundeni Clinical Institute, Bucharest, Romania; 4. Expert Center for Rare Haematological Disorders, Sofia, Bulgaria; 5. National Haemophilia Centre and Haemostasis Department, Medical Centre of Hungarian Defence Forces, Budapest, Hungary; 6. Department of Haematology, University Medical Centre Ljubljana, Faculty of Medicine, University of Ljubljana, Ljubljana, Slovenia; 7. Department of Clinical Haematology, University Hospital Brno, Brno, Czech Republic; 8. Department of Laboratory Methods, Faculty of Medicine, Masaryk University, Brno, Czech Republic; 9. National Research Center for Hematology, Moscow, Russia; 10. Department of Hemostasis Disorders and Internal Medicine, Institute of Hematology and Transfusion Medicine, Warsaw, Poland

**Keywords:** hemophilia, extended half-life, prophylaxis, ABR – annualized bleed rate, aPCC – activated prothrombin complex concentrates, BPAs – bypassing agents, BU – Bethesda units, EHL – extended half-life, FVIII/IX – factor VIII/IX, ITI – immune tolerance induction, mHJHS – modified Haemophilia Joint Health Score, pd – plasma-derived, PPX – prophylaxis, QoL – quality of life, r – recombinant, rFVIIIFc – recombinant FVIII Fc fusion protein, rFVIIIpeg – recombinant pegylated FVIII, SHL – standard half-life, siRNA – small interfering RNA, TFPI – tissue factor pathway inhibitor, TGA – thrombin generation assays, WFH – World Federation of Hemophilia

## Abstract

The next frontier in hemophilia A management has arrived. However, questions remain regarding the broader applicability of new and emerging hemophilia A therapies, such as the long-term safety and efficacy of non-factor therapies and optimal regimens for individual patients. With an ever-evolving clinical landscape, it is imperative for physicians to understand how available and future hemophilia A therapies could potentially be integrated into real-life clinical practice to improve patient outcomes. Against this background, nine hemophilia experts from Central European countries participated in a pre-advisory board meeting survey. The survey comprised 11 multiple-choice questions about current treatment practices and future factor and non-factor replacement therapies. The survey questions were developed to reflect current unmet needs in hemophilia management reflected in the literature. The experts also took part in a follow-up advisory board meeting to discuss the most important unmet needs for hemophilia management as well as the pre-meeting survey results. All experts highlighted the challenge of maintaining optimal trough levels with prophylaxis as their most pressing concern. Targeting trough levels of ≥30–50 IU/L or even higher to achieve less bleeding was highlighted as their preferred strategy. However, the experts had an equal opinion on how this could be achieved (*i.e.*, more efficacious non-factor therapies or factor therapy offering broader personalization possibilities such as targeting trough levels to individual pharmacokinetic data). In summary, our study favors personalized prophylaxis to individual pharmacokinetic data rather than a "one-size-fits-all" approach to hemophilia A management to maintain optimal trough levels for individual patients.

## Introduction

Patients with hemophilia have historically been treated by replacement of the deficient coagulation factor using either plasma-derived (pd) or recombinant (r) factor VIII/IX (FVIII/IX) replacement products [1–3]. One of the major limitations of factor concentrates is the need for multiple intravenous access at least two to three times weekly for prophylaxis (PPX) and/or on-demand intravenous administration to treat acute bleeding [1–3]. In recent years, several strategies to reduce treatment burden by extending the half-life of factor concentrates have led to the development and approval of seven recombinant extended half-life (EHL) products [4, 5]. Yet, despite effective PPX, the development of allo-antibodies (inhibitors) to infused coagulation factor continues to be the most frequent and serious complication in the management of severe hemophilia, leading to an increased risk of difficult-to-treat bleeding [4]. Bypassing agents (BPAs) such as recombinant factor VIIa (rFVIIa) and activated prothrombin complex concentrates (aPCC) are standard treatments to circumvent factor use to treat acute bleeding in individuals with high-titer or high-responding inhibitors (≥5 Bethesda units BU/mL), but these agents are associated with inconsistent predictability in terms of efficacy [6, 7]. For example, rFVIIa is effective in 70–100% of mild to severe bleeding episodes with high-responding inhibitors, with better results achievable when used early [8]. 

In 2018, the first non-factor replacement therapy, emicizumab, was approved for use in Europe for long-term PPX in people of all ages who have congenital hemophilia A with FVIII inhibitors or severe congenital hemophilia A (FVIII<1%) without FVIII inhibitors [4, 9, 10]. Emicizumab, a bispecific monoclonal antibody that mimics the activity of FVIII by binding activated factors IX and X, maintains a level of hemostatic activity estimated at 9–10% of FVIII activity [11, 12] and thus offers the potential for a clinically meaningful reduction of bleeding episodes in patients with hemophilia A who have developed inhibitors compared to on-demand/prophylactic use of BPAs [4, 9, 10]. Its weekly subcutaneous dosing schedule has been reported to provide health-related quality of life (QoL) and health status benefits [9, 13]. However, unprovoked breakthrough bleeding may still occur, plus emicizumab is insufficient on its own to prevent bleeding in the setting of trauma or major surgery, necessitating treatment with other BPAs such as rFVIIa and aPCC [1, 9,13–16]. Breakthrough bleeding may also still occur with emicizumab therapy, and serious thromboembolic safety concerns with concomitant aPCC have been observed in the early phase 3 HAVEN-1 trial [1, 9, 13–15]. Therefore, further data are required to establish the long-term safety and efficacy outcomes of emicizumab [17]. Furthermore, it has been suggested that eradicating inhibitors via immune tolerance induction (ITI) rather than treating suboptimally with large quantities of hemostatic agents in hemophilia patients with life-long inhibitors may be a preferred strategy from a cost-effectiveness and long-term societal perspective [18].

Despite these remarkable advances in hemophilia management over the past decade, challenges remain, including breakthrough bleeding, progressive joint disease, inhibitor development, lower efficacy of BPAs in inhibitor patients compared with FVIII/FIX replacement therapy in non-inhibitor patients, and QoL aspects. Several innovative pharmacological agents with unique mechanisms of action have shown the potential to alleviate some of the current challenges presented with existing factor replacement products by rebalancing hemostasis in people with hemophilia A and B [7, 19]. Efanesoctocog alfa (BIVV001), a novel fusion protein designed to overcome the von Willebrand factor-imposed half-life ceiling and maintain high sustained factor VIII activity levels [20], was granted fast-track designation in the US for the treatment of hemophilia A [21]. Non-factor strategies in addition to emicizumab include subcutaneous small interfering RNA (siRNA) prophylactic therapy to lower antithrombin levels [7, 19, 22] and humanized monoclonal antibodies that target the tissue factor pathway inhibitor (TFPI) [23, 24]. Gene therapy aiming for phenotypic cure is also being evaluated [7, 25].

The updated World Federation of Hemophilia (WFH) 2020 guidelines reflect the latest developments in the management of hemophilia [5]. PPX, redefined by the WFH, is considered the standard of care for patients with hemophilia A and B [5]. The PPX treatment paradigm has shifted from simply increasing factor levels to maintain a trough factor level of 1% towards allowing people with hemophilia to lead healthy and active lives [5]. Importantly, the WFH recognizes the need for physicians to support higher trough levels (3–5%) to achieve PPX and introduces steady-state hemostasis as a possible new target for PPX [5]. Moreover, personalized PPX that takes into account patient self-assessment and preference is also advised [5].

Physicians now face a plethora of challenging choices for factor replacement, so it is important to understand how unmet needs in the management of people with hemophilia can be addressed in the future. To this end, we summarize the results of a pre-meeting survey and follow-up discussions from an advisory board held by a group of hemophilia experts to gain valuable insights into how new and emerging therapies may help improve outcomes and QoL through individualized PPX and maintenance of appropriate trough levels.

## Material and Methods

Invitations from Sobi™, Switzerland, according to the latest communication guidelines, were sent to nine expert hemophilia opinion leaders from Central European countries (Bulgaria, Croatia, Czech Republic, Hungary, Poland, Romania, Russia, Slovakia, and Slovenia) to participate in a pre-meeting survey and follow-up advisory board meeting. All nine (100%) invited experts agreed to participate in the pre-meeting survey, which took place between 14^th^–21^st^ September 2021. Sobi developed survey questions and response options about existing and future factor and non-factor replacement therapies to reflect current unmet needs in hemophilia management identified in the literature. The final 11 multiple-choice questions were: (1) What laboratory assays are available in your practice? (2) How do challenges in laboratory monitoring of different therapies using different assays impact your therapy decision pathway in hemophilia A? (3) Reflecting your clinical practice, which parameters have you observed when introducing/switching a person with hemophilia to an EHL product, *e.g.*, recombinant FVIII Fc fusion protein (rFVIIIFc), from a standard half-life (SHL) (pdFVIII or rFVIII) product? (4) Have you experienced neutralizing antibody formation in previously treated hemophilia A patients without inhibitors on prophylaxis in your clinical practice? (5) Do you experience an increased interest in non-factor replacement therapeutics among hemophilia A patients without inhibitors? (6) Based on your clinical experience and available data from the literature, what is your opinion on the maintenance of joint health with factor and non-factor therapies? (7) In your opinion, what is the efficacy of factor *versus* non-factor therapy when aiming for 3–5% trough in hemophilia A prophylaxis? (8) What would you expect if factor prophylaxis was intensified to aim for 10% trough in hemophilia A? (9) In your opinion, which future PPX therapies have the potential to become mainstream choices for people with hemophilia A without inhibitors? (10) If EHL FVIII concentrate was available at the same payer price as SHL FVIII, would you consider switching a well-controlled person with hemophilia A to EHL? and (11) In your opinion, how can QoL be improved in the future for people with hemophilia A? These questions were distributed and anonymously collected through the web-based application Survey Monkey (SurveyMonkey, San Mateo, CA, USA). Since the survey did not measure specific constructs, no psychometric testing or content validity was conducted. No incentives were offered for participation in the survey, and no expert could review and change the given answers after submission. All results were analyzed using descriptive statistics, and no formal statistical analysis was performed.

The virtual follow-up advisory board meeting was held on 21^st^ September 2021, which provided an opportunity for the experts to discuss the pre-meeting survey results.

## Results

### Insights into hemophilia treaters' current clinical practice

**Figure 1.**
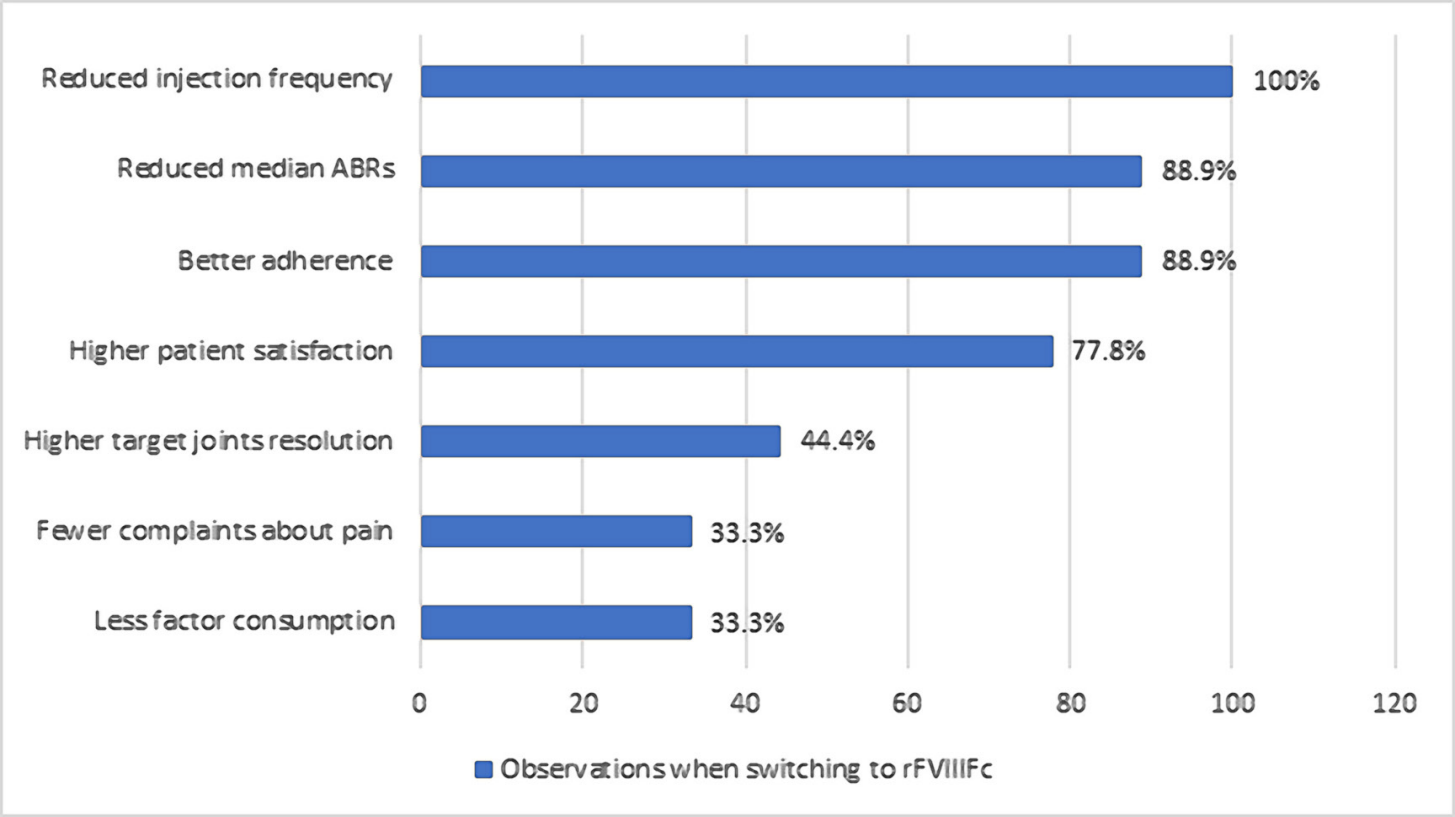
Question: "Reflecting your clinical practice, which of the following have you observed when introducing/switching to EHL (e.g., rFVIIIFc) from SHL (pdFVIII or rFVIII) products?". Experts selected all options that applied. ABR – annualized bleeding rate; EHL – extended half-life; rFVIII – recombinant factor VIII; rFVIIIFc – rFVIII fusion protein; pdFVIII – plasma-derived factor VIII; SHL – standard half-life.

The experts were asked about the different laboratory assays available in their practice. All nine experts had access to the traditional one-stage clotting assay to quantify FVIII activity to classify disease severity or monitor treatment. In addition, FVIII chromogenic assays that use bovine- or human-based components and thrombin-generation assays are available in the respective laboratories of more than 50% of the experts. First, the experts were asked: "How do challenges in laboratory monitoring of different therapies using different assays impact your therapy decision pathway in hemophilia A". For the majority of the experts (77.8%), difficulties in laboratory monitoring did have somewhat of an impact (44.4%) or was a real obstacle (33.3%) affecting their treatment decision when choosing prophylaxis for a person with hemophilia A. Next, the experts were asked about their clinical observations when introducing/switching a person with hemophilia to an EHL product, *e.g.*, recombinant FVIII Fc fusion protein (rFVIIIFc), from a standard half-life (SHL) (pdFVIII or rFVIII) product (Figure 1). Eight experts indicated that they had experienced better median annualized bleed rates (ABRs), improved adherence, and higher patient satisfaction (Figure 1). However, only one-third of the experts indicated that they had experienced their patients on EHL prophylaxis consuming less factor concentrate and complaining less about joint pain.

One-third of the experts stated that they had no experience of inhibitor formation in previously treated hemophilia A patients without inhibitors on PPX in their clinical practice. In addition, the experts indicated that inhibitor formation in previously treated children with hemophilia on any type of PPX is rare. Notably, the experts reported no inhibitor formation for any previously treated patient on EHL products, rFVIIIFc or recombinant pegylated FVIII (rFVIIIpeg), or the non-factor replacement product, emicizumab; note: anti-emicizumab antibodies, including some with emicizumab-neutralizing activity, have been reported in a few people with hemophilia A treated with emicizumab [26–28]. 

Next, the experts were questioned about interest in non-factor replacement therapeutics among hemophilia patients without inhibitors. People with hemophilia with a high intravenous infusion burden were most likely to seek information about non-factor products (66.7%), followed by patients with regular bleeds (3.3%). However, two out of nine experts (22.2%) indicated that non-factor therapy was not yet available in their country for patients with hemophilia without inhibitors.

**Figure 2.**
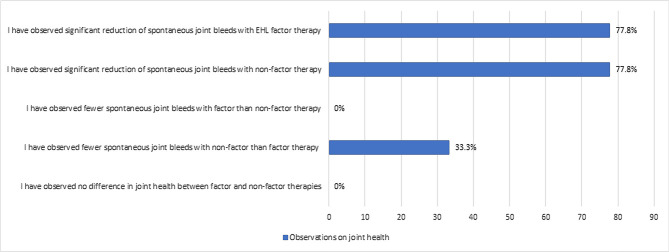
Question: "Based on your clinical experience and available data from the literature, what is your opinion on maintenance of joint health with factor and non-factor therapies?". Experts selected all options that applied. EHL – extended half-life.

The experts participating in the survey were asked their opinion on maintaining joint health with factor and non-factor therapies. An equal number of the experts indicated that they had observed a significant reduction of spontaneous joint bleeds in patients on both non-factor and EHL factor PPX (Figure 2). One-third of experts indicated that they had observed fewer spontaneous joint bleeds with non-factor therapy than with factor therapy (Figure 2); however, as mentioned above, two of the experts had no experience with non-factor therapy as it is unavailable in their respective countries, so they would have agreed/disagreed with this statement based on available evidence from the literature.

### Insights into experts' opinion of existing prophylaxis therapies

**Figure 3.**
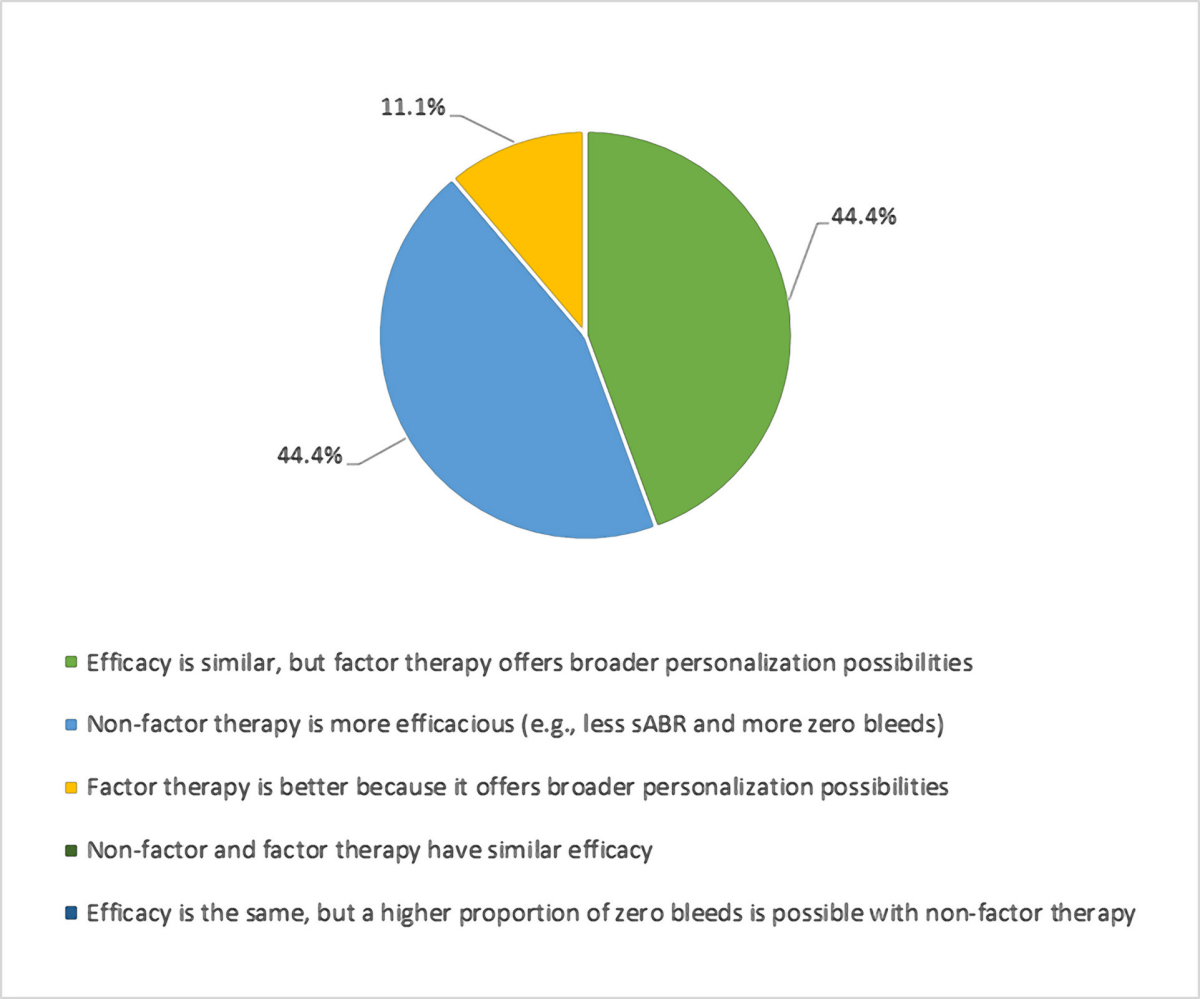
Question: "In your opinion, is factor or non-factor therapy more efficacious when aiming for 3–5% trough in hemophilia A prophylaxis?". sABR – spontaneous annualized bleeding rate.

**Figure 4.**
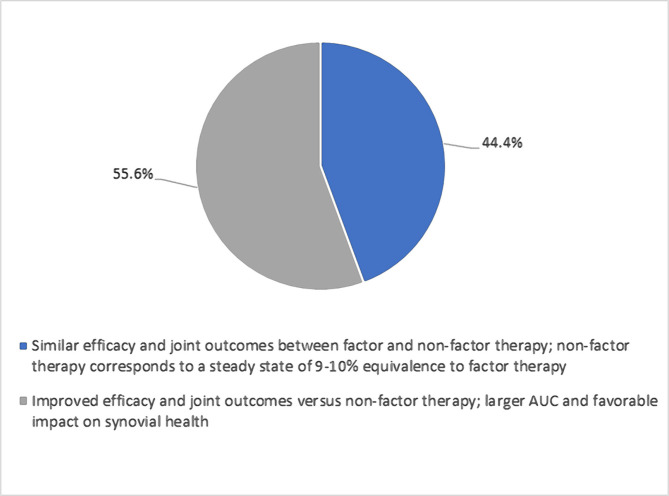
Question: "What would you expect if factor prophylaxis in hemophilia A is intensified to aim for 10% trough?". AUC – area under curve.

Based on personal experience, the experts were asked their opinion on the efficacy between factor and non-factor replacement therapy when aiming for a target 3–5% trough in hemophilia A patients on PPX. Opinions differed, with an equal split between the number of experts indicating that non-factor therapy was more efficacious and those that stated factor therapy offered broader personalization possibilities, *e.g.*, targeting trough levels to individual pharmacokinetic data, level and timing of physical activity, condition of their musculoskeletal system etc, in addition to similar efficacy to non-factor therapy (Figure 3). Similarly, there was a split in opinion about whether factor therapy offered similar or improved efficacy to non-factor therapy if prophylaxis was to be intensified to aim for a 10% trough in hemophilia A (Figure 4).

### Insights into experts' opinion of future prophylaxis therapies

When asked about future PPX in hemophilia patients without inhibitors, the majority of the experts (88.9%) agreed that the new class of FVIII replacement therapy, namely efanesoctocog alfa (BIVV001), shows promise to become a mainstream option because it has the potential for more optimal, extended protection against all bleeding types in patients with severe hemophilia A, as compared with other hemostasis rebalancing therapies and bispecific monoclonal antibodies such as emicizumab. In addition, the experts unanimously agreed that if EHL FVIII concentrate was available at the same payer price as SHL FVIII, they would consider switching well-controlled patients in their practice to EHL, mainly due to the ability to attain higher trough levels.

**Figure 5.**
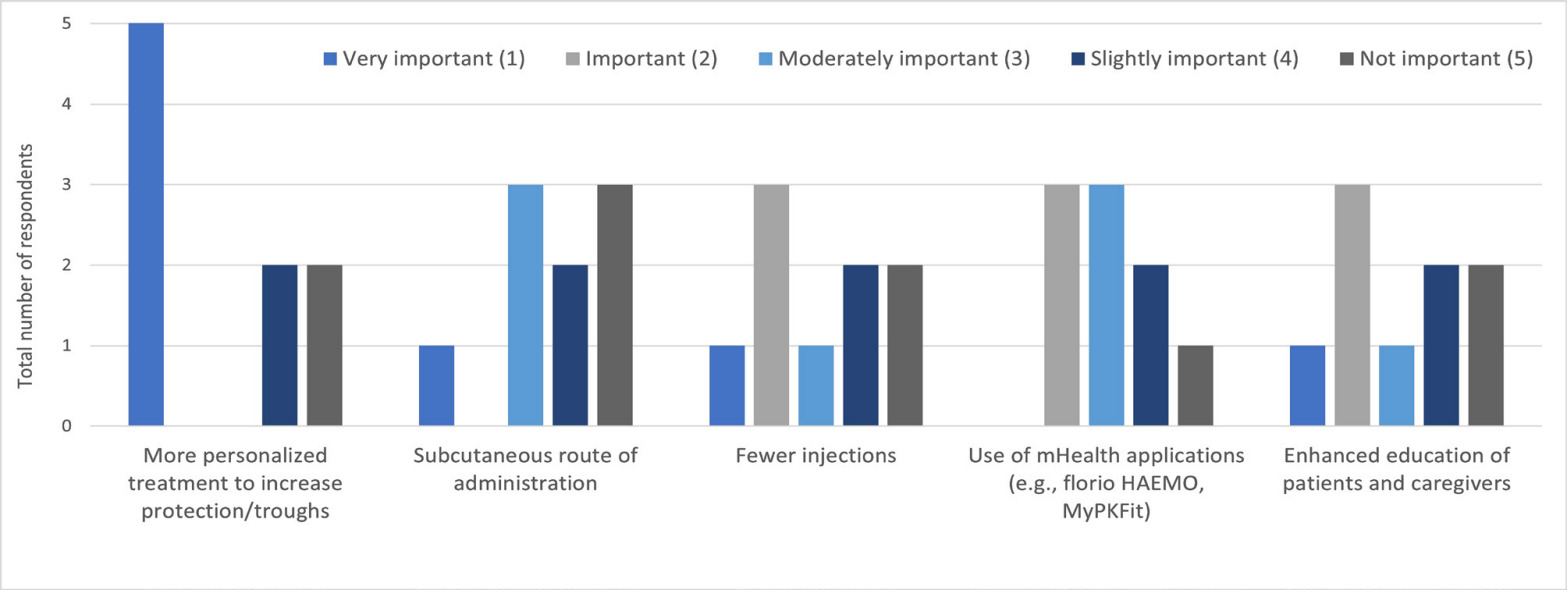
Question: "In your opinion, how can quality of life (QoL) be improved for people with hemophilia A, ranking 1–5, 1 being most important?".

The experts were asked their opinion on how QoL could be improved in the future for people with hemophilia. More than 50% of the experts felt that more personalized treatment to increase protection was very important, and the use of telemedicine applications such as florio^®^ HAEMO (Sobi) and MyPKFit™ (Takeda) was important/very important (Figure 5). On the other hand, subcutaneous route of administration, fewer injections, and enhanced education of patients and caregivers were considered less important strategies for improving QoL.

## Discussion

New and emerging therapies such as novel EHL factor concentrates and non-factor treatments will likely reshape hemophilia care within the next decade, providing more efficacious and convenient management options and possibly curative therapies. To gain further insight into how these new treatments could potentially be integrated into real-life clinical practice to improve patient outcomes, hemophilia experts from nine countries across Central Europe took part in a pre-meeting survey and follow-up discussions. During the advisory board discussions, it was noted that the most recent controversy in the hemophilia community concerns the optimal trough level for PPX. 

In patients with severe hemophilia, PPX was traditionally considered the standard of care with the goal of treatment to maintain factor levels well above 1% at all times, based on the conventional aim to prevent joint bleeds and maintain musculoskeletal health [5, 29]. However, there is increasing recognition and evidence from the literature that factor trough levels of 1–3% are insufficient to prevent bleeds in all patients with hemophilia [5]. It has also been suggested that maintaining higher factor levels (above 10%) may be optimal to prevent subclinical bleeding and the gradual progression of joint disease over a lifespan in very active patients [30]. The WFH has subsequently redefined PPX based on patient outcome measures rather than therapeutic doses or treatment regimen initiation time as the routine administration of a hemostatic agent to prevent bleeding in people with hemophilia while allowing them to maintain an active lifestyle and achieve QoL comparable to individuals without hemophilia [5]. If the new treatment aim for PPX is to increase the trough level, existing and future prophylactic regimens are likely to require adjustment [5]. Maintaining such high trough levels in some patients may lead to the re-shortening of longer treatment intervals and, consequently, an increase of previously reduced factor consumption, which has been an important benefit of EHL products [30]. The WFH guidelines recommend using emicizumab PPX as one possible option to prevent hemarthrosis, both spontaneous and breakthrough bleeding, in patients with severe hemophilia A without inhibitors [5]. rFVIIIFc is strongly equivalent to emicizumab in terms of mean ABR and proportion of patients with zero bleeds when considering a conservative approach to target factor levels (1–3% trough) and aiming for troughs of 3–5%; however, factor therapy offers more personalization possibilities compared with emiciaumab [5, 31]. Moreover, serious thromboses and thrombotic microangiopathy episodes associated with the concomitant use of aPCC were observed in HAVEN-1, which led to emicizumab having a special warning regarding its concomitant use with aPCC [9, 13]. Emicizumab treatment also has the potential for immunogenicity, albeit with a low neutralizing potential (<1% patients) [16, 32]. Therefore, there is an unmet need for additional, novel therapies with long-term safety and efficacy data.

During the follow-up advisory board discussion, the nine experts discussed their preference(s) to use therapies that target higher trough levels between 3–5% or even higher to achieve less bleeding for their hemophilia patients. The experts also discussed the need to treat very active hemophilia A patients requiring higher trough levels (5–10%) with EHL rFVIII PPX rather than non-factor therapies. However, the experts noted that trough levels are just one aspect of patients' care, and long-term clinical outcome data needs to be collected to see whether such an approach can improve care.

EHL products prolong the half-life of recombinant coagulation factors and allow a lower annual burden of intravenous injections than SHL factor concentrates [4, 33]. Moreover, higher trough levels of FVIII (2–3%) can be achieved with EHL than with SHL products [33–35]. A lower annual consumption in units of EHL products has also been reported in clinical trials [34, 35]. The real-life experience of most of the experts in our survey was that for patients switching from SHL to EHL products, there was a reduction in injection frequency, better median ABRs, improved adherence, and higher patient satisfaction. In addition, one-third of the experts reported that their patients had consumed fewer EHL units than the previous SHL product. If costs were similar between SHL and EHL products, most hemophilia experts in our survey stated that they would consider switching their well-controlled patients to EHL products to attain higher trough levels.

One of the most challenging complications in hemophilia A treatment is the development of neutralizing anti-FVIII antibodies, with up to approximately 30% of previously treated patients with severe hemophilia A developing inhibitors [36–38]. Consistent with data from clinical trials with EHL products [34, 39], the nine experts in our survey observed no inhibitor formation in real-life clinical practice to date for their previously treated hemophilia A patients on EHL products (rFVIIIFc or rFVIIIpeg) or emicizumab.

Because FVIII/FIX level is strongly correlated with disease severity, hematologists have relied on factor level as the key determinant of bleeding risk. Indeed, our survey shows that all nine representative countries rely on the one-stage clotting assay. The advent of EHL products and novel non-factor therapies, *i.e.*, emicizumab, subcutaneous siRNA prophylactic therapies such as fitusiran, and anti-tissue factor pathway inhibitors such as marstacimab, may lead to new challenges in laboratory monitoring of patients [40]. Notably, each of the latter therapies has a different mechanism of action and requires a different monitoring approach and assay [40]. Thrombin generation assays (TGA) are "global" assays measuring the dynamics of the blood coagulation process beyond endpoint assays [41]. TGA has demonstrated a significant potential for monitoring the efficacy of PPX for various established and novel hemophilia therapies, including non-factor therapies [41]. Yet, the use of global assays with non-factor therapies such as emicizumab in the clinical setting is still in its infancy [41]. Just over half of the experts in our survey reported having access to two-stage chromogenic clotting assays that can be used to measure the functional activity of FVIII/IX or TGAs. Furthermore, most experts agreed that the complexity and difficulty in monitoring new innovative therapies would significantly impact treatment decisions. In order to enable personalized treatment for hemophilia patients, it will be necessary for specialized clinical laboratories to be fully equipped with the required equipment, product-specific reagents, and expertise to perform appropriate assays and monitor levels of coagulation activity [40]. However, the option to prescribe newer therapies such as emicizumab is not available in all the countries represented by the experts in our survey yet.

Recurrent hemarthrosis leads to joint damage and hemophilic arthropathy, increasing morbidity and decreasing QoL [5]. Improvements in joint health have been observed over time in hemophilia patients receiving EHL PPX, as assessed using the modified Haemophilia Joint Health Score (mHJHS) [42]. Similarly, with pooled data for long-term emicizumab PPX, low ABRs are maintained and bleeding into target joints decreases substantially with no new occurrences [43]. In real-life clinical practice, our survey highlights that EHL factor and non-factor PPX effectively reduce spontaneous joint bleeds. However, the experts agreed that careful, regular monitoring of joint status is required to enable early intervention to prevent arthropathy. Non-factor prophylactic therapies are not associated with the peak and trough curves of protection typically observed with factor prophylaxis regimens. Although most patients on emicizumab do not bleed, the experts agreed that this does not exclude residual arthropathy, disability and chronic pain, which could be avoided with factor peaks. Furthermore, experimental evidence suggests that factor VIII plays a role in maintaining skeletal health [44, 45]. Therefore, caution is required in prescribing newer therapies and long-term follow-up is required to understand more about outcomes with emicizumab and other novel non-factor therapies. 

Increased protection is an important trend, primarily achievable through individualized PPX, education, EHL factor concentrates, and electronic diary tools such as HaemoAssist^®^ 2, Haemtrack, Smart Medication^®^, myPKFiT^®^ and, the most recently introduced app, florio^®^ HAEMO [46–49]. Calculating dose and dosing frequency based on individual pharmacokinetic (PK) response to factor VIII (FVIII) infusions is the WFH approach for personalizing prophylactic regimens [5]. When asked about strategies to improve QoL for patients in the future, more than half of the experts agreed that personalized treatment to increase protection is very important, and telemedicine applications are "very important" or "important". In addition, most of the experts agreed that novel therapies such as the new class of FVIII replacement therapy, efanesoctocog alfa (BIVV001), would become another mainstream therapy due to its potential to achieve personalized, extended protection against all bleeding types in patients with severe hemophilia A.

This study has limitations. Given the small number of selected experts who completed the survey, the opinions are unlikely to represent the entire hemophilia community. It must also be kept in mind that this particular survey was conducted in the context of a highly dynamic and changing treatment landscape, and as new clinical data become available, expert opinion may change over time. The survey questions and response options were developed by Sobi, Switzerland, to best reflect current unmet needs in hemophilia management identified in the literature; however, no content validity was performed to reduce the risk of bias. Although the survey was conducted prior to the advisory board discussions, it may be possible that the industry affiliations of some authors led to unmindful response bias. During the follow-up discussions, it was noted that the survey question relating to QoL did not capture all elements that might impact QoL, *e.g.*, improving mobility, reducing pain or anxiety, allowing a more unrestricted life etc.

Further, some aspects of this survey depended on experts' opinions, experience, and memory, as well as the availability of products in the represented countries, which may have influenced responses and/or introduced elements of recall bias. Finally, other inherent limitations relate to the cross-sectional nature of the survey. Therefore, the findings of this survey should be interpreted in light of the limitations mentioned above.

## Conclusions

EHL recombinant factor products and non-factor therapies such as emicizumab are used in clinical practice. Many more new therapies are at various stages of clinical development. How these new therapies will be integrated into real-life clinical practice and long-term outcome data of recently introduced treatment modalities remain to be defined at a later stage. For factor PPX, targeting trough levels of at least 3–5% or even higher to achieve less bleeding is now a widely accepted treatment strategy. Factor PPX may be preferable for active hemophilia A patients rather than non-factor therapy since it offers broader personalization possibilities. However, maintaining such high trough levels may require a re-shortening of longer treatment intervals, which has been one of the most important benefits of available EHL products to date; this creates space for next-generation FVIII replacement therapy.

In contrast to the peak and trough curves of protection typically observed with factor prophylaxis regimens, newer therapies such as emicizumab reduce bleeding risk but do not necessarily exclude residual arthropathy, disability, and chronic pain. Therefore, these newer therapies should be used with caution until long-term follow-up data become available. Our study supports individualized prophylaxis rather than a "one-size-fits-all" approach to attain optimal trough levels for each patient, as endorsed by the WFH 2020 guidelines.

## Acknowledgments

### Conflict of interest

ABat received research funding from Novo Nordisk, Octapharma and Sobi, honoraria for consultancy and/or speakers fees from CSL Behring, Novo Nordisk, Octapharma, Sobi, Roche, and Takeda; ABob received honoraria as a member of advisory board and/or speaker from Bayer, CSL Behring, Octapharma, Pfizer, Novo Nordisk, Roche, Takeda and Sobi; TL received research funding from Bayer, CSL Behring, Catalist, Grifols, Octapharma, Pfizer, Sanofi-Alnilam and Bioverative, honoraria for advisory board participation from Roche and Sobi, and lecture fees from Novo Nordisk, Roche and Sobi; LN received consulting fees and honoraria for lectures from CSL Behring, Sobi, Roche, Pfizer, Takeda, Novo Nordisk and Bayer; IPZ received speaker fees from Bayer, Pfizer, Novo Nordisk, Roche, Takeda and Sobi; PS received lecture fees from CSL Behring, NovoNordisk, Octapharma, Roche, Sobi and Takeda; JW received grant support from Alnylam Pharmaceuticals, LFB, Novo Nordisk, Octapharma, Rigel Pharmaceuticals, Roche, Shire/Takeda and Sobi, lecture fees from Alexion, Baxalta, CSL Behring, Ferring Pharmaceuticals, Novo Nordisk, Octapharma, Roche, Sanofi/Genzyme, Shire/Takeda, Siemens, Sobi and Werfen; NZ and MB declare no conflicts of interest.

### Ethical approval

Ethical approval for completing the survey or participation in the discussion meeting was not requested since no identifying data were collected, and consent was assumed by participating in the survey and attending the online meeting.

### Funding

Sobi (Switzerland) funded this survey.

### Personal thanks

We thank Dr. Klara J. Belzar and XRL8 Health Ltd., Hertfordshire, UK, for medical writing support of the manuscript (including writing, language editing, referencing, formatting, and proofreading), which was funded by Sobi (Switzerland). 

### Authorship

AB, AB, MB, TL, LN, IPZ, PS, NZ, JW equally contributed to the conceptualization and methodology of the study and reviewing the manuscript.
